# Editorial: Advancements in Molecular Diagnosis and Treatment of Melanoma

**DOI:** 10.3389/fonc.2021.728113

**Published:** 2021-07-09

**Authors:** Giuseppe Palmieri, Igor Puzanov, Daniela Massi, Paolo Antonio Ascierto

**Affiliations:** ^1^ University of Sassari & Unit of Cancer Genetics, National Research Council (CNR), Sassari, Italy; ^2^ Roswell Park Comprehensive Cancer Center, University of Buffalo, Buffalo, NY, United States; ^3^ Section of Pathology, Department of Health Sciences, University of Florence, Florence, Italy; ^4^ Melanoma, Cancer Immunotherapy and Development Therapeutics Unit, Istituto Nazionale Tumori Fondazione G. Pascale, Naples, Italy

**Keywords:** melanoma, molecular diagnosis, cancer progression, targeted therapy, immunotherapy

Melanoma is characterized by a marked molecular heterogeneity, considerably greater than that highlighted so far from the histopathological and clinical points of view only. The development and progression of melanoma, like almost all other forms of malignant neoplasms, is based on the acquisition of sequential alterations in specific gene pathways or metabolic/molecular mechanisms involved in the regulation of cell functions **(** 1, 2 **)**.

The role of genetic and epigenetic alterations in the onset and progression of tumors is being steadily established. Intracellular alterations occurring in molecular pathways have been found to even concur in interfering with the homeostasis of the tumor microenvironment (TME). As consequence, a tight interaction between intracellular changes and various extracellular factors participating in immune activity against the tumor is strongly involved in modulating neoplastic progression. One can summarize that cancer cells develop and progress under the pressure of an articulated network of intra- and extracellular growth stimuli.

In this complex scenario, several TME elements are progressively taking the stage: immune cells (including, in addition to the main effectors such as CD8+/CD4+ T lymphocytes and natural killer cells, a whole system of cells with regulatory and immunosuppressive activity), endothelial cells and vascular changes aimed at increasing angiogenesis, fibroblasts, components of the extracellular matrix (including those involved in epithelial-mesenchymal transition and/or stroma remodeling), and a variety of soluble molecules (such as growth factors, chemotactic factors, cytokines, etc.). On this latter aspect, several conditions may tip the scales in favor of an immunosuppressive or an immune reactive status. Here is a tentative list of examples of such conditions: altered levels of VEGF, interleukins, immune checkpoint effectors, cyto/chemokines, or enzymes such as IDO and arginase; variation of the TME concentration of immunosuppressive cells such as myeloid-derived suppressor cell/MDSC, tumor-associated macrophage/TAM, or regulatory T cell/Treg; unbalanced distribution of dendritic/mature dendritic cells **(** 3 **)**. Overall, these elements form a complex regulatory network that favors tumor growth by creating an environment that allows tumor cells to evade immune surveillance.

Among the events intrinsic to the cells, some molecular alterations may be able to profoundly affect the tumor sensitivity to T lymphocyte activity and, more in general, the capability of exerting an antitumor immune reaction. Again, here is an indicative and not fully comprehensive list of such molecular changes: silencing of PTEN, MAPK activation, enhanced PI3K activity, activated WNT/β-catenin signaling, JAK1/2 inactivating mutations, STAT1-3/STING/TBK1 signaling impairment, increased rates of chromosomal instability or aneuploidy, modifications in antigen/neoantigen presentation **(** 3 **)**.

During the last decade, a real revolution has been registered for both management and treatment of melanoma. Before 2010, only one fourth of patients with metastatic disease were alive at 1 year **(** 4 **)**. Current therapeutic strategies allowed the achievement of outstanding results represented by high response rates and prolonged disease control; to date, about half of patients with advanced melanoma is indeed alive at 5 years **(** 5 **)**. Predominantly, therapeutic strategies that have contributed in recent years to change the outcome of melanoma patients with subsequent significant impact on long-term benefit include either a selective blocking of the BRAF-driven signal transduction (BRAF mutant inhibitors—vemurafenib, dabrafenib, encorafenib—given in combination with MEK inhibitors—cobimetinib, trametinib, binimetinib) either the immune checkpoint blockade therapy (targeting CTLA-4—ipilimumab—and the PD-1/PD-L1 axis—nivolumab, pembrolizumab). Moreover, the continuously improved experience of clinicians in managing sequence or combination of the above mentioned therapies as well as in appropriately integrating systemic treatments with specific loco-regional interventions (i.e. radiotherapy, metastasectomy, electrochemotherapy, etc.) significantly increased the chances of prolonged survival in ever larger groups of melanoma patients **(** 5 **)**.

Unfortunately, the failure of the disease control into the remaining half of melanoma patients, who thus progress to death in the same time period, represents the disappointing other side of the coin. In this regard, several studies are ongoing in order to either investigate new treatment protocols either optimize the strategies for the best use of the currently available drugs. Trials are being conducted for defining the most effective sequence of targeted and immune checkpoint therapy in *BRAF*-mutated melanoma patients - also trying to clarify whether translational studies may be helpful in selecting distinct subsets of responders and non responders - as well as for determining the most appropriate regimens to be used after progression to the first-line treatments **(** 6 **)**. Continuous efforts to strengthen the integration of surgical and medical interventions are likely to be the key in improving long-term outcomes in patients with melanoma.

The Research Topic “Advancements in molecular diagnosis and treatment of melanoma” provides an overview of the main strategic approaches aimed at improving the clinical benefits for the different patients’ subsets, by including:

- identification of additional molecular pathways and new available drugs, also considering preclinical and clinical data available for several targets under development **(** 7 **)**. This will pave the way for further investigations on modalities of combining them with existing targeted or immune therapies as well as on evaluation of the safety and tolerability of such combination or sequential therapies;
- development of methods capable of predicting patient response or resistance to different systemic treatment options (mostly, immunotherapy) by mainly providing circulating tumor-derived elements as non-invasive biomarkers (so-called “liquid biopsy”). In this sense, a clinical practice change into the management of melanoma patients would be represented by a “dynamic” characterization of the (epi)genetic and molecular signatures, to be assessed not only at baseline but also during the course of treatment or follow-up. In other words, the aim should be to monitor any biological variation of the disease behavior depending on intrinsic and acquired tumor heterogeneity **(** 8 **)**;
- assessment of the right time for therapy administration, when treatments may exert their maximal clinical benefit in terms of rates of patients alive with no evidence of disease (adjuvant and neoadjuvant approaches);
- identification and translation into the clinical practice of deeper mutational profiling driven by new artificial intelligence tools (i.e. digitalization of tissue slides for recognizing all melanoma features to standardize diagnoses or better classify tumor microenvironment components as well as use of faster software for interpretation of multi-parametric data and development of bioinformatic algorithms). This would more accurately weight the specific contribution of any molecular feature to the disease behavior, in a patient-matched way;
- methodological improvement of single-cell testing and multiplexed immunohistochemical or transcriptional assays for a more detailed evaluation of the functional roles of the genes associated with melanoma, toward a better understanding of their prognostic and/or predictive significance.

## Author Contributions

GP wrote the Editorial. IP, DM, and PAA helped in discussing the content. All authors contributed to the article and approved the submitted version.

## Funding

We thank the Fondazione AIRC “Programma di ricerca 5 per Mille 2018-ID#21073” for partially funding this work.

## Conflict of Interest

The authors declare that the research was conducted in the absence of any commercial or financial relationships that could be construed as a potential conflict of interest.
